# Quantum technology for development framework as a tool for science diplomacy

**DOI:** 10.3389/frma.2023.1279376

**Published:** 2023-12-19

**Authors:** Mhlambululi Mafu, Makhamisa Senekane

**Affiliations:** ^1^Department of Physics, Case Western Reserve University, Cleveland, OH, United States; ^2^Institute for Intelligent Systems, University of Johannesburg, Johannesburg, South Africa; ^3^National Institute for Theoretical and Computational Sciences, Johannesburg, South Africa

**Keywords:** quantum diplomacy, quantum technology, Global North, Global South, Sustainable Development Goals

## Abstract

The state-of-the-art quantum technologies leverage the unique principles of quantum mechanics, which include quantization, uncertainty principle, interference, entanglement and decoherence, to produce useful devices and scientific advancements not possible with classical technologies. As a result, quantum technologies, in particular, offer specific advantages that make communications networks secure and unbreakable and devices with unprecedented levels of accuracy, responsiveness, reliability, scalability and efficiency than classical emerging technologies. These capabilities can contribute significantly to addressing energy, agriculture, climate change, national security, healthcare, education and economic growth challenges. Unfortunately, these developments in these areas have not been evenly distributed between the Global North and the Global South, inadvertently creating a societal and economic gap. Closing this gap is critical to creating a more inclusive and sustainable future for all, thus delivering key sustainable goals. Therefore, to close this gap, this article proposes a quantum diplomacy framework as a means to deliver science diplomacy. Moreover, we discuss how emerging quantum technologies could profoundly impact all 17 United Nations Sustainable Development Goals. We consider this work a timely and vital intervention to prevent the gap from increasing.

## 1 Introduction

The United Nations (UN) has declared 2020 to 2030 the decade of action and delivery for the Sustainable Development Goals (SDGs) (UNGA, 2019). The SDGs are 17 global goals that were adopted by the UN Member States in 2015 as a universal call for action by all countries to end poverty and other deprivations that must go hand-in-hand with strategies that improve health and education, reduce inequality, and spur economic growth-all while tackling climate change and working to preserve our oceans and forests.^1^ To leave no one behind, these goals must be pursued in an integrated manner (O'Sullivan, 2021). Notably, these goals have far-reaching and transformative implications requiring concerted effort from the government, businesses, and individuals worldwide to address the world's social, economic, and environmental challenges. These implications embrace global collaboration, policy integration, inclusive development, monitoring and accountability, private sector engagement, sustainable consumption and production, behavioral changes and climate action. Therefore, by embracing these implications, the world can work toward a more equitable, prosperous, and sustainable future for everyone. As a result, within the context of quantum technology (QT), we broadly explore how the proposed Quantum Technology for Development (QT4D) framework can effectively advance these implications.

The framework of Information and Communications Technology for Development (ICT4D) and Artificial Intelligence for Development (AI4D) have been used previously to deliver the Sustainable Development Goals (SDGs) (Unwin, 2009; Walsham, 2017). Since ICT and AI are directly related to QT (Singh et al., 2020; Aithal, 2023), it can be seen as another unique technological tool to deliver the SDGs. Fundamentally, QT is a field encompassing technology that solely relies on the unique properties of quantum mechanics to deliver devices and process information in a manner that offers advantages over current conventional technology, which can be understood within the framework of classical mechanics (Nielsen, 2002). Generally, QT is driven by two imperatives: practicality, which drives miniaturization, which has become a dominant trend in technological innovation, and fundamentality, where quantum mechanics provides unmatched performance over what can be achieved in classical frameworks (Dowling, 2003). This is called “quantum supremacy”, which is not simply about how fast and more powerful the quantum technologies are but rather about solving complex problems beyond the capacity or design of classical technologies. Essentially, there are four main QT categories: quantum communication, quantum computing, quantum metrology and sensing (Thew, 2019). Most significantly, these QT fields have become a key enabler of several emerging technologies, with applications in sports (Torgler, 2020), biology (Marais et al., 2018), chemistry (Lanyon et al., 2010; Deglmann et al., 2015), nuclear physics (Carlson et al., 2015, 2018), chemical engineering (Ajagekar and You, 2022), business and finance (Orús et al., 2019; Aljaafari, 2023), education (Fox et al., 2020), healthcare (Ur Rasool et al., 2023), agriculture (Wang and Blagrave, 2021), cybersecurity and defense (Krelina, 2021) and, surprisingly, the social sciences and humanities, for instance, psychology (Busemeyer and Wang, 2015). Therefore, this broad influence of QT signals that, in addition to being a fundamental technological enabler that could impact every sector (Jaeger, 2018), it has significant potential to revolutionize how countries conduct international relations and science diplomacy. Furthermore, this demonstrates that current and emerging quantum technologies could have substantial social and economic competitiveness and geopolitical impact.

Globally, QT investment is about $30 billion and is expected to be $42.4 billion by 2027 [Oxford University Innovation (2014)]. This demonstrates commitment and understanding of QT's strategic potential and illustrates the massive cost of investing in QT infrastructure. Unfortunately, these programs are absent or inadequately coordinated in the Global South. Since these advances reflect a promising future, the prevailing power dynamics have resulted in the critical uneven adoption and distribution of quantum technologies between the Global North and the Global South (Reuveny, 2008). Some barriers to adoption are due to regulatory, technological, talent acquisition, identification of practical use cases and financial (Al Natsheh et al., 2015; Krishnakumar, 2020). Moreover, while the Global North has recognized the need for cooperation to share advances and has signed various agreements, this contrasts with the Global South. This has exacerbated existing inequalities and created new ones. For instance, in most Global South countries, societal and economic development has unevenly resulted in social unrest, political instability, economic stagnation and unending intellectual migration to the Global North countries. This is an opportune moment to close this gap for all societies to flourish in the modern world. As a result, we propose a QT4D to catalyze effective science diplomacy between the Global North and the Global South, including the Global South-South. The QT4D is based on the premise that QT can significantly address global development challenges. Specifically, science diplomacy uses diplomatic assistance to foster scientific cooperation and exchange to promote international relations (Xuereb, 2022). While this concept is relatively new, QT's accelerated development and impact have become increasingly crucial in transforming modern society and international collaboration among scientists, policymakers, and industry partners. Thus, we leverage the QT4D to promote international cooperation and collaboration driven by diplomatic assistance. The main research questions or objectives of our study are:

1. What can QT4D do to minimize the gap between the Global North and the Global South?
2. Why quantum technologies, as a specific example of emerging technologies have a potential to deliver science diplomacy between Global North and South?
3. How can QT address global challenges such as climate change, energy sustainability, and improved healthcare and food security?

The Global South has a long history of manufacturing and QT dating back to the 19th century (Reuveny, 2008). For instance, Satyendra Nath Bose co-discovered bosons, a fundamental particle in quantum mechanics, with Albert Einstein [The Nobel Prize (1979)]. Sheldon Lee Glashow and Steven Weinberg also shared the 1979 Nobel Prize for Physics with Abdus Salam for their contribution to electroweak unification theory [ICTP (1964)]. Abdus Salam played a critical role in establishing the International Centre for Theoretical Physics in Italy, which has trained thousands of Global South scientists. In 1988, Marcos Moshinsky Borodiansky, a Mexican, won the Prince of Asturias Prize for Scientific and Technical Investigation and 1997 the UNESCO Science Prize in recognition of his work on elementary particles [ICTP-SAIFR (2019)]. Further, countries in the Global South, such as Brazil, Chile, and South Africa, have significantly contributed to quantum physics (Forbes, 2021). Despite these achievements, technology advancement has shifted unevenly toward the North over the past few decades. As a result, there has been an ever-increasing QT gap and uneven adoption of sophisticated resources between the Global North and the Global South, leading to prevailing power dynamics that are likely to continue (Jang, 2022).

**Figure 1:**
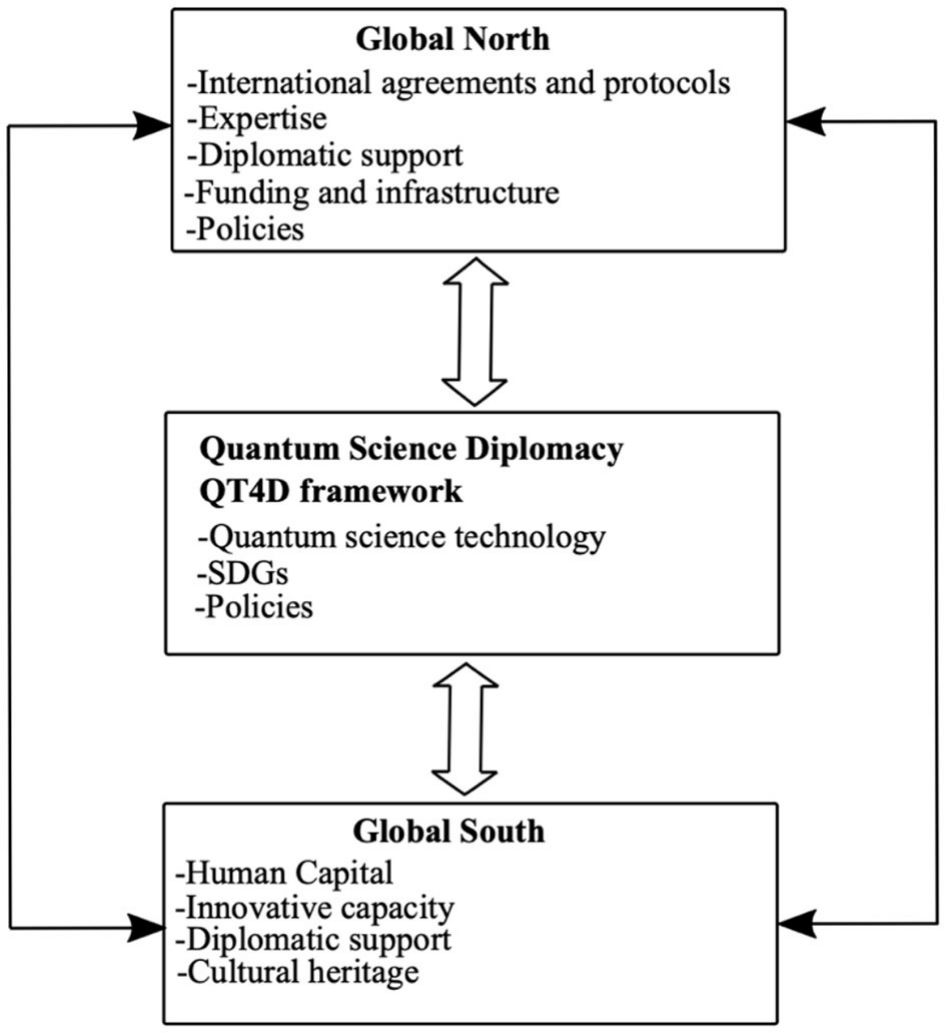
Conceptual framework for fostering collaborations between the Global North and Global South.

**Figure 2:**
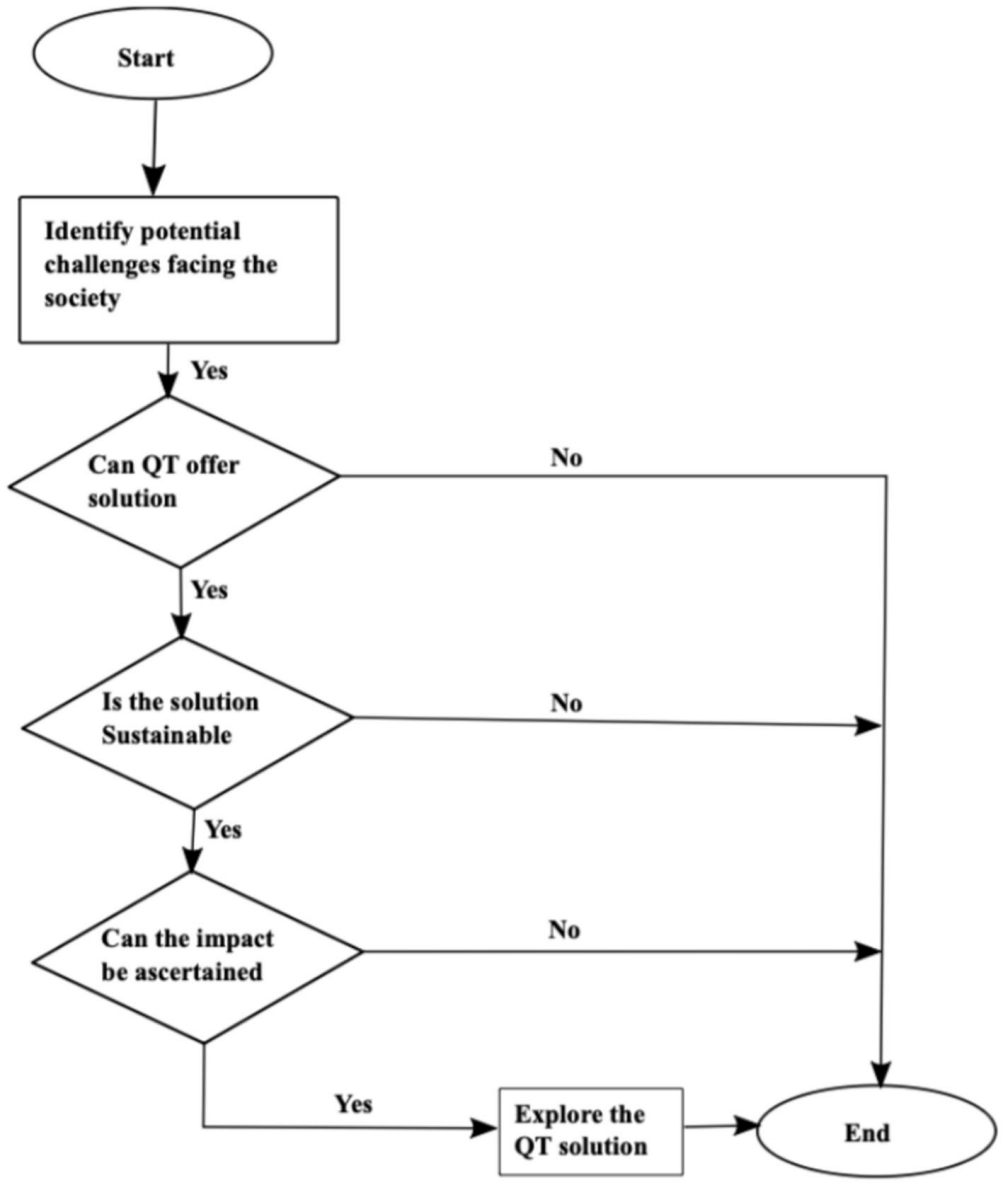
A schematic diagram of the proposed QT4D framework.

Figure 1 illustrates the concept framework we developed to demonstrate how quantum diplomacy could bridge the technology gap between the Global North and the Global South. The Global North countries have the most sophisticated technologies, including QTs. By utilizing science diplomacy, the proposed QT4D and the primary elements, including quantum science technologies, science diplomacy, SDGs, and policies, could close the gap. We will discuss how some of these elements could impact collaborative activities. The highly specialized nature of QT challenges the full adoption of sophisticated technologies in the Global South. The Global South must train, retain, and attract the best talent to participate effectively in the second quantum revolution. Furthermore, QT academic and research programs remain underfunded in the Global South. Additionally, intellectual property awareness and expertise could be higher, partly due to a lack of effective national systems supporting technology transfer and commercialization. As a tool to drive science diplomacy between the Global North and the Global South, we propose the QT4D framework shown in Figure 2.

## 2 Quantum technology: applications and potential driver to deliver science diplomacy

QTs, which encompass a wide range of scientific advancements with no classical analog, have emerged as prime examples of emerging technologies that hold a significant potential to revolutionize various fields, including science and diplomacy (Bjola, 2016; Degelsegger-Márquez, 2020). Specifically, through harnessing the unique properties of quantum mechanics, QTs offer innovative solutions to complex problems and have a potential to address several global challenges (Acín et al., 2018), hence bridge the gap between Global North and South and contribute to the SDGs. Below we provide reasons why QTs have the potential to deliver science diplomacy and summarize use cases in Table 1.

SDG 1–2 and SDGs 11–15: Quantum computers use superposition and entanglement to perform complex computations more efficiently or “exponentially” faster than classical computers (Steane, 1998). The unprecedented computing power and capability make it possible to solve complex optimization problems and analyze multiple datasets simultaneously. Most significantly, quantum computers can efficiently process and learn quantum algorithms, offering novel approaches to machine learning tasks such as optimizing the training of deep neural networks, enabling them to learn complex pattens more effectively. Hence, quantum computers can analyze big data more efficiently, which allows conclusions to be drawn more efficiently and predictions to be made more accurately (Chen and Zhang, 2014; Mallow et al., 2022). As a result, quantum computers could be used to model climate and optimize agricultural processes, such as weather forecasting and crop management, leading to increased crop yields and food security. Additionally, quantum computers can monitor financial markets in real-time, enabling traders to make informed investment decisions in a fraction of the time required by classical computers (Orús et al., 2019).
SDG 3: QTs have the potential to revolutionize healthcare and medicine (Batool et al., 2022; Ur Rasool et al., 2023). Quantum sensors can detect and diagnose diseases with unprecedented sensitivity (Tarasov et al., 2020). This enables early detection and diagnosis of diseases. Moreover, quantum computing can simulate complex biological systems, leading to the development of new drugs and therapies (Cao et al., 2018). QTs can provide secure transmission of medical data, ensuring privacy (Senekane et al., 2017; Senekane, 2019) and confidentiality (Garapo et al., 2016; Mafu and Senekane, 2018).
SDG 4 and SDG 8: Quantum machine learning (QML) algorithms can improve the accuracy of machine learning (ML) tasks by incorporating quantum algorithms and data structures (Mafu and Senekane, 2021; Caro et al., 2022). Thus, using QML, educational institutions can analyze a vast amount of data (Ciliberto et al., 2018). This data could include student performance, attendance records, and demographic information, to identify students at risk of dropping out or struggling academically. Moreover, this data can relate to employees requiring upskilling or further development. An important point to note is that identifying at-risk students and people requiring further training or upskilling is a complex task that requires careful consideration of several factors. Classical ML algorithms may have difficulties handling the complexities of educational data, such as nonlinear relationships and noisy data. QML can overcome these limitations by providing enhanced computing power and quantum-inspired algorithms. Thus, QML is poised to revolutionize the field of educational analytics or predictive learning analytics (Mafu, 2023) and provide targeted support to students in need worldwide.
SDG 5 and SDG 10: QT can significantly impact the algorithms and decision-making process of AI systems. AI models trained on historical data often exhibit inherent biases, resulting in discriminatory outcomes (Mehrabi et al., 2021). The powerful computational capabilities of quantum computers can overcome these limitations. A quantum algorithm can analyze large datasets more efficiently and accurately, allowing it to identify and resolve biases (Bharti et al., 2022). Therefore, quantum computers can provide alternative solutions to complex problems, offering more diverse and unbiased results. This could include applications in healthcare, finance, and criminal justice industries, where bias can have significant consequences. This implies, QT can foster global inclusivity, reduce disparities, and connect remote areas. As a result, QT has the potential to revolutionize the pursuit of inclusive and equitable representation in various sectors of society. Overall, this will promote diversity, equity, equality and inclusion.
SDG 6: Quantum sensors operate by applying the principles of quantum mechanics to improve their sensitivity and accuracy (Dowling, 2003; Degen et al., 2017; Jaeger, 2018). Thus, they operate at the quantum level, where classical physics rules cease to apply. There are two kinds of quantum sensors: photonic quantum sensors, which are based on the interaction of light with matter, and quantum mechanical sensors, which use quantum phenomena to detect and quantify physical quantities. Due to their high levels of precision, these sensors are ideal for applications that require high levels of accuracy, such as metrology, spectroscopy, and remote sensing (Mukamel et al., 2020; Yin et al., 2020). As a result, quantum sensors have several advantages when identifying water sources since they feature high sensitivity, wideband detection, non-invasive and non-destructive techniques, real-time monitoring, and long-term monitoring and maintenance of water sources. Due to this, as QT continues to evolve, they have the potential to revolutionize the field of water resource science and engineering. Therefore, QT can be a valuable tool for addressing water scarcity and promoting sustainable water management in the Global North and South.
SDG 7: Quantum computers can simulate complex chemical reactions (Brown et al., 2010) while quantum sensors can accurately monitor and optimize energy production systems (Polymeni et al., 2023). This leads to more efficient utilization of resources and sustainable energy solutions, which can benefit both regions since the Global North strives for more efficient and sustainable energy solutions. At the same time, the Global South often struggles to access reliable and clean energy sources. As a result, working together will facilitate the development of innovative solutions with both economic and environmental benefits.
SDG 3, SDG 9, and SDG 16: Quantum cryptography provides authentication and unbreakable encryption or unconditional security over traditional cryptographic methods by exploiting the principles of quantum mechanics, such as superposition and entanglement (Gisin et al., 2002; Mafu and Senekane, 2018). Thus, it offers a secure way to exchange sensitive information, ensuring the confidentiality of sensitive data relating to finance, healthcare, and government communications. Besides its security benefits, quantum cryptography also offers scalability (Pirandola et al., 2020). Thus, it has the potential to support extensive networks, allowing for secure communication over a global scale, thus ensuring data protection and combat cyber-terrorism. Moreover, scalability is critical for applications such as IoT and cloud computing (Hua et al., 2023), which could enhance communication and connectivity, promote economic growth and job creation, foster collaboration, and scientific research, contribute to sustainable development, and bridge divides.

**Table 1:** The proposed QT4D potential use cases.

**SDG**	**Potential use case**
SDG1	Quantum analysis using quantum computers
SDG2	Quantum machine learning to predict climate-resistant crops Quantum computers for analysis of big data for land use, agricultural drought Quantum sensing for imagery Quantum machine learning for yield forecasting
SDG3	Quantum computers for health big data analytics Quantum cryptography for health data protection Quantum machine learning for drug discovery
SDG4	Quantum machine learning to identify at-risk students Equitable QT training (skills development)
SDG5	Inclusive and equitable representation in QT Female-friendly QT environment
SDG6	Quantum sensing to identify water sources Quantum dots to test water quality
SDG7	Quantum machine learning for energy forecasting
SDG8	Intensive QT up-skilling
SDG9	Innovation in QT QT industrialization and entrepreneurship for inclusive job creation Fair and equitable access to QT infrastructure
SDG10	Inclusive access to QT tools
SDG11	Quantum computers for big data analytics
SDG12	Quantum computers for big data analytics
SDG13	Quantum computers for climate modeling
SDG14	Quantum computers for big data analytics
SDG15	Quantum computers for big data analytics
SDG16	Quantum cryptography to combat cyber-terrorism
SDG17	Strategic partnerships for equitable access to QT resources; ensuring that no one is left behind

This illustrates how utilizing the appropriate quantum technology described could be used to achieve the 17 SDGs described in Figure 2. This provides a correlation between each of the 17 SDGs and the quantum technologies that could assist in achieving those goals.

SDGs, Sustainable Development Goals; QT, Quantum Technology.

## 3 Quantum technology for development (QT4D) framework

The QT4D framework is an innovative tool that aims to leverage the potential of QT and facilitate its deployment and implementation to address global challenges and contribute toward achieving the SDGs through science diplomacy. The QT4D framework offers a unique platform to enhance access to QTs in the Global South by creating opportunities for knowledge sharing and international collaboration, which will lead to alignment of research interests and identification of common goals, increased capacity building, and facilitating implementation of innovative quantum-based solutions. Thus, by integrating this framework with science diplomacy, the Global North and South can unlock quantum technologies' full potential, foster coordinated cooperation, and ensure no one is left behind. According to Figure 2, we first identify a developmental challenge facing society. Therefore, the next step is identifying the QT to be used to address the developmental challenge. This is followed by assessing whether the QT solution is sustainable. If the QT solution is sustainable, the next step assesses the impact of the QT solution. Finally, if such a solution is impactful, a partnership is formed through science diplomacy to explore the QT solution.

Through diplomatic-assisted collaboration among governments, the private sector, and academic institutions, the QT4D framework provides an enabling environment for QT development in the Global South. Furthermore, the QT4D framework could facilitate South-South cooperation between the QT haves and have-nots in the Global South. Since most countries in the Global South face challenges in cybersecurity, healthcare, climate change, energy sustainability, and agriculture (Acharya, 2016), QTs such as quantum communication, quantum computing, sensing, and materials could provide cutting-edge solutions to these challenges. The QT4D framework can deliver improved healthcare in the Global South by delivering QTs with applications in precision medicine and accurate diagnostic tools and fostering access to healthcare professionals and personalized, affordable treatments. For instance, quantum sensors and imaging can aid in early disease detection (Matea et al., 2017; Tarasov et al., 2020). Quantum communication through quantum cryptography (Gisin et al., 2002; Mafu and Senekane, 2018) could ensure that diplomatic dialogues remain confidential, fostering improved candid discussions and trust and strengthening international relations. Quantum computing is suited to analyze large datasets (Mallow et al., 2022) and identify new drug targets (Cao et al., 2018). This capability will enable personalized treatment options based on the analysis of large datasets to identify patterns to create more effective treatments for diseases such as malaria and tuberculosis, develop accurate drug simulations, enabling Global North and South researchers to create more effective drugs. Furthermore, quantum computing could enable diplomats to analyze vast amounts of data at unprecedented speeds, make data-driven decisions, and engage in complex negotiations more effectively.

Most Global South countries still face challenges in generating sustainable energy solutions for their populations (Hostettler, 2015). QTs can transform the energy sector, offering new clean energy generation, transmission, and storage solutions. Quantum sensors can monitor and measure energy consumption patterns, while quantum power plants can generate clean and renewable energy more efficiently. Moreover, the Global South is home to a substantial proportion of the world's population who rely on agriculture for their livelihoods and face challenges in security (Carlson, 2018). Moreover, QT4D can provide a platform for quantum sensor development. Sensors monitor and measure environmental factors, including air, water, and soil quality. They can also assist in monitoring crops and detecting nutrient deficiencies, which lead to higher crop yields and enhanced food security, including alleviating the effects of climate change. On the other hand, quantum sensing holds great promise for diplomacy. Quantum sensors can detect and sense phenomena at unprecedented sensitivity levels, opening up new possibilities for surveillance and security (Degen et al., 2017). This technology can be implemented in various areas, such as border control, cybersecurity, and defense. By leveraging quantum sensing, nations can improve their ability to detect and deter potential threats, ensuring the security of their citizens and diplomatic personnel. Regarding information security, QTs can provide enhanced cybersecurity solutions with cryptography, encryption and authentication applications. Quantum computing can potentially revolutionize the Global South's agricultural production levels. For instance, due to quantum computing, food supply chains can be optimized, food waste reduced, and countries in the Global South can make informed decisions about managing natural resources and mitigating climate change through real-time data. As a result, food production levels in the Global South will likely match those in the Global North.

Notably, there are further aspects the Global South can offer quantum diplomacy, such as their distinct histories, cultures, and geopolitical circumstances (Papastergiadis, 2017). By utilizing these insights, quantum diplomacy can address specific global challenges like climate change and social inequality with valuable insights. Moreover, quantum computing technology already exists in several Global South countries, a diverse talent pool, and an entirely different perspective on diplomacy. Science, technology, engineering, and mathematics (STEM) workers in many Global South countries are highly skilled and highly educated, making them well-positioned to shape quantum diplomacy in the future. For example, Brazil, India, and South Africa, already have access to quantum computing (Forbes, 2021). Through this expertise, valuable insights and perspectives can be gained regarding the use of QTs for diplomatic purposes, as well as training to work with QTs. By utilizing these resources, we can develop a more inclusive and equitable framework for quantum diplomacy by providing insight into QT's ethical and societal implications. On the other hand, most of the Global North countries have developed solid diplomatic relations worldwide (Lancaster, 2008). Through the partnerships, a team of quantum scientists could be assembled to conduct active research engagements, develop promising technology cases for industry, hire quantum data scientists, and redesign reskilling programs. Moreover, diplomatic support can assist the Global South in offering specialized post-graduate QT degree programs to bridge the skills gap, attract top talent, create employment opportunities, and contribute to economic growth. Another technological contribution realizes that the “quantum internet”, one of QT's primary goals (Kimble, 2008), will ensure ultra-secure telecommunications and facilitate effective communication between communities in the Global South and the Global North. Ultimately, this has a significant potential to level all societal and economic challenges, eradicating the digital divide and bringing all societies together.

## 4 Discussion and conclusion

As we conclude, this work provides a novel approach to utilizing QTs to facilitate science diplomacy, two fields that have traditionally operated independently. Precisely, we demonstrate how advances in QT can potentially be leveraged to foster diplomatic-assisted international collaboration and cooperation in scientific research and promote sustainable global development in a mutually beneficial manner to achieve the 17 SDGs. Most significantly, while QT continues gaining traction due to its disruptive potential in various fields such as quantum communication, quantum computing, quantum metrology, and sensing, its application within science diplomacy goes beyond its technical aspects and has only received limited exploration. Thus, the QT4D framework recognizes the importance of bridging this gap, particularly by exploring opportunities to address global challenges such as climate change, energy sustainability, healthcare, and agriculture. Other benefits include bridging political, cultural, and geographical boundaries. As a result, the proposed framework has practical implementations and significance in integrating scientific research, policy formulation, and global cooperation, providing a roadmap for the Global North and South to harness QT effectively. Most significantly, it is essential to note that even though the QT4D framework exhibits considerable utility in fostering science diplomacy to advance science, it still faces some inherent challenges, such as the limited availability of quantum infrastructure (quantum devices, quantum laboratories, and research centers), skilled personnel or quantum workforce, countries exploiting QTs for strategic advantage and possible misuse of QT for military purposes leading to the arms race, preventing its full adoption. However, the QT4D framework can serve as a platform for establishing norms and guidelines for the responsible development and implementation of QTs. Moreover, this work provides the foundation for future advancements in these two traditionally inherent fields, contributing to the broader 17 SDGs, science diplomacy, and global cooperation. Finally, this work opens intriguing possibilities for exploration and discussion in quantum diplomacy.

## Data availability statement

The original contributions presented in the study are included in the article/supplementary material, further inquiries can be directed to the corresponding author.

## Author contributions

MM: Conceptualization, Formal analysis, Methodology, Project administration, Supervision, Writing – original draft, Writing – review & editing. MS: Conceptualization, Formal analysis, Funding acquisition, Writing – original draft, Writing – review & editing.

## Footnotes

^1^ https://sdgs.un.org/goals

## References

[B1] Acharya A. (2016). Advancing global IR: Challenges, contentions, and contributions. Int. Stud. Rev. 18, 4–15. 10.1093/isr/viv016

[B2] Acín A. Bloch I. Buhrman H. Calarco T. Eichler C. Eisert J.. (2018). The quantum technologies roadmap: a European community view. New J. Phys. 20, 080201. 10.1088/1367-2630/aad1ea

[B3] Aithal P. S. (2023). Advances and new research opportunities in quantum computing technology by integrating it with other ICCT underlying technologies. Int. J. Case Stud. Bus. IT Educ. 7, 314–358. 10.47992/IJCSBE.2581.6942.0304

[B4] Ajagekar A. You F. (2022). New frontiers of quantum computing in chemical engineering. Korean J. Chem. Eng. 39, 811–820. 10.1007/s11814-021-1027-6

[B5] Al Natsheh A. Gbadegeshin S. A. Rimpiläinen A. Imamovic-Tokalic I. Zambrano A. (2015). Identifying the challenges in commercializing high technology: a case study of quantum key distribution technology. Technol. Innov. Manag. Rev. 5, 26–36. 10.22215/timreview/864

[B6] Aljaafari M. (2023). Quantum computing for social business optimization: a practitioner's perspective. Soft Comp. 1–23. 10.1007/s00500-023-08764-y

[B7] Batool S. Nabipour H. Ramakrishna S. Mozafari M. (2022). Nanotechnology and quantum science enabled advances in neurological medical applications: diagnostics and treatments. Med. Biol. Eng. Comput. 60, 3341–3356. 10.1007/s11517-022-02664-3 36207564

[B8] Bharti K. Cervera-Lierta A. Kyaw T. H. Haug T. Alperin-Lea S. Anand A.. (2022). Noisy intermediate-scale quantum algorithms. Rev. Mod. Phys. 94, 015004. 10.1103/RevModPhys.94.015004

[B9] Bjola C. (2016). Getting digital diplomacy right: what quantum theory can teach us about measuring impact. Global Affairs 2, 345–353. 10.1080/23340460.2016.1239388

[B10] Brown K. L. Munro W. J. Kendon V. M. (2010). Using quantum computers for quantum simulation. Entropy. 12, 2268–2307. 10.3390/e12112268

[B11] Busemeyer J. R. Wang Z. (2015). What is quantum cognition, and how is it applied to psychology? Curr. Dir. Psychol. Sci. 24, 163–169. 10.1177/0963721414568663

[B12] Cao Y. Romero J. Aspuru-Guzik A. (2018). Potential of quantum computing for drug discovery. IBM J. Res. Dev. 62, 6–1. 10.1147/JRD.2018.2888987

[B13] Carlson C. (2018). Rethinking the agrarian question: agriculture and underdevelopment in the Global South. J. Agrarian Change 18, 703–721. 10.1111/joac.12258

[B14] Carlson J. Dean D. J. Hjorth-Jensen M. Kaplan D. Preskill J. Roche K.. (2018). Quantum Computing for Theoretical Nuclear Physics, a White Paper Prepared for the US Department of Energy, Office of Science, Office of Nuclear Physics. Washington, DC: USDOE Office of Science (SC) (United States).

[B15] Carlson J. Gandolfi S. Pederiva F. Pieper S. C. Schiavilla R. Schmidt K. E.. (2015). Quantum Monte Carlo methods for nuclear physics. Rev. Mod. Phys. 87, 1067. 10.1103/RevModPhys.87.1067

[B16] Caro M. C. Huang H. Y. Cerezo M. Sharma K. Sornborger A. Cincio L.. (2022). Generalization in quantum machine learning from few training data. Nat. Commun. 13, 4919. 10.1038/s41467-022-32550-3 35995777 PMC9395350

[B17] Chen C. P. Zhang C. Y. (2014). Data-intensive applications, challenges, techniques and technologies: a survey on Big Data. Inf. Sci. 275, 314–347. 10.1016/j.ins.2014.01.015

[B18] Ciliberto C. Herbster M. Ialongo A. D. Pontil M. Rocchetto A. Severini S.. (2018). Quantum machine learning: a classical perspective. Proc. R. Soc. Mat. Phy. Eng. Sci. 474, 20170551. 10.1098/rspa.2017.0551 29434508 PMC5806018

[B19] Degelsegger-Márquez A. (2020). “International dimensions of the EU's FET Flagships: large-scale strategic research investments as a site of de-facto science diplomacy,” in Science Diplomacy in the Making: Case-Based Insights From the S4D4C Project, 116. Available online at: https://www.s4d4c.eu/wp-content/uploads/2020/03/D3.2_5_FET_Flagships_final.pdf

[B20] Degen C. L. Reinhard F. Cappellaro P. (2017). Quantum sensing. Rev. Mod. Phys. 89, 035002. 10.1103/RevModPhys.89.035002

[B21] Deglmann P. Schäfer A. Lennartz C. (2015). Application of quantum calculations in the chemical industry—an overview. Int. J. Quantum Chem. 115, 107–136. 10.1002/qua.24811

[B22] Dowling JP Milburn GJ (2003). Quantum technology: the second quantum revolution. Philos Trans A Math Phys Eng Sci. 361, 1655–1674. 10.1098/rsta.2003.1227 12952679

[B23] Forbes A Petruccione F Roux FS (2021). Toward a quantum future for South Africa. AVS Quant. Sci. 3, 040501. 10.1116/5.0060426

[B24] Fox M. F. Zwickl B. M. Lewandowski H. J. (2020). Preparing for the quantum revolution: what is the role of higher education? Phy. Rev. Phy. Educ. Res. 16, 020131. 10.1103/PhysRevPhysEducRes.16.020131

[B25] Garapo K. Mafu M. Petruccione F. (2016). Intercept-resend attack on six-state quantum key distribution over collective-rotation noise channels. Chin. Phy. B 25, 070303. 10.1088/1674-1056/25/7/070303

[B26] Gisin N. Ribordy G. Tittel W. Zbinden H. (2002). Quantum cryptography. Rev. Mod. Phys. 74, 145. 10.1103/RevModPhys.74.145 10990784

[B27] Hostettler S. (2015). “Energy challenges in the Global South,” in Sustainable Access to Energy in the Global South: Essential Technologies and Implementation Approaches, (Cham: Springer International Publishing), 3–9.

[B28] Hua X. Li D. Fu Y. Zhu Y. Jiang Y. Zhou J.. (2023). Hierarchical controlled hybrid quantum communication based on six-qubit entangled states in IoT. Sensors. 23, 9111. 10.3390/s23229111 38005499 PMC10674261

[B29] ICTP (1964). Our History. Available online at: https://www.ictp.it/home/our-history (accessed August 3, 2023).

[B30] ICTP-SAIFR (2019). ICTP-SAIFRRoundtable on Quantum Computing and its Applications. Available online at: https://www.ictp-saifr.org/roundqc/ (accessed June 19, 2023).

[B31] Jaeger L. (2018). The Second Quantum Revolution. Switzerland: Springer.

[B32] Jang B Choung J-Y Kang I (2022). Knowledge production patterns of China and the US: quantum technology. Scientometrics 127, 5691–5719. 10.1007/s11192-022-04478-4

[B33] Kimble H. J. (2008). The quantum internet. Nature 453, 1023–1030. 10.1038/nature07127 18563153

[B34] Krelina M. (2021). Quantum technology for military applications. EPJ Quant. Technol. 8, 24. 10.1140/epjqt/s40507-021-00113-y

[B35] Krishnakumar A. (2020). Quantum Computing and Blockchain in Business: Exploring the Applications, Challenges, and Collision of Quantum Computing and Blockchain. Birmingham: Packt Publishing Ltd.

[B36] Lancaster C. (2008). Foreign Aid: Diplomacy, Development, Domestic Politics. Chicago, IL: University of Chicago Press.

[B37] Lanyon B. P. Whitfield J. D. Gillett G. G. Goggin M. E. Almeida M. P. Kassal I.. (2010). Towards quantum chemistry on a quantum computer. Nat. Chem. 2, 106–111. 10.1038/nchem.483 21124400

[B38] Mafu M. (2023). “Leveraging disruptive technologies and systems thinking approach at higher education institutions,” in The Sustainable University of the Future: Reimagining Higher Education and Research (Cham: Springer International Publishing), 25–42.

[B39] Mafu M. Senekane M. (2018). “Security of quantum key distribution protocols,” in Advanced Technologies of Quantum Key Distribution (London: IntechOpen).

[B40] Mafu M. Senekane M. (2021). “Design and Implementation of Efficient Quantum Support Vector Machine,” in 2021 International Conference on Electrical, Computer and Energy Technologies (ICECET) (Piscataway, NJ: IEEE), 1–4.

[B41] Mallow G. M. Hornung A. Barajas J. N. Rudisill S. S. An H. S. Samartzis D. (2022). Quantum computing: the future of big data and artificial intelligence in spine. Spine Surg. Rel. Res. 6, 93–98. 10.22603/ssrr.2021-0251 35478980 PMC8995124

[B42] Marais A. Adams B. Ringsmuth A. K. Ferretti M. Gruber J. M. Hendrikx R.. (2018). The future of quantum biology. J. R. Soc. Int. 15, 20180640 10.1098/rsif.2018.0640 PMC6283985 30429265

[B43] Matea C. T. Mocan T. Tabaran F. Pop T. Mosteanu O. Puia C.. (2017). Quantum dots in imaging, drug delivery and sensor applications. Int. J. Nanomedicine 5421–5431. 10.2147/IJN.S138624 28814860 PMC5546783

[B44] Mehrabi N. Morstatter F. Saxena N. Lerman K. Galstyan A. (2021). A survey on bias and fairness in machine learning. ACM Comp. Surv. 54, 1–35. 10.1145/3457607

[B45] Mukamel S. Freyberger M. Schleich W. Bellini M. Zavatta A. Leuchs G.. (2020). Roadmap on quantum light spectroscopy. J. Phy B Atom. Mol. Opt. Phy. 53, 072002. 10.1088/1361-6455/ab69a8

[B46] Nielsen MA, and Chuang, I.. (2002). Quantum Computation and Quantum Information. New York, NY: Cambridge University Press.

[B47] Orús R. Mugel S. Lizaso E. (2019). Quantum computing for finance: overview and prospects. Rev. Phy. 4, 100028. 10.1016/j.revip.2019.100028

[B48] O'Sullivan K Clark S Marshall K MacLachlan M (2021). A just digital framework to ensure equitable achievement of the sustainable development goals. Nat. Commun. 12, 6345. 10.1038/s41467-021-26217-8 34732699 PMC8566573

[B49] Oxford University Innovation (2014). Taking a Leap Towards the Quantum Future. Available online at: https://innovation.ox.ac.uk/wp-content/uploads/2016/02/Taking-a-leap-towards-the-quantum-future.pdf (accessed July 27, 2023).

[B50] Papastergiadis N. (2017). The end of the Global South and the cultures of the South. Thesis Eleven 142, 69–90. 10.1177/0725513617712790

[B51] Pirandola S. Andersen U. L. Banchi L. Berta M. Bunandar D. Colbeck R.. (2020). Advances in quantum cryptography. Adv. Optics Photon. 12, 1012–1236. 10.1364/AOP.361502

[B52] Polymeni S. Plastras S. Skoutas D. N. Kormentzas G. Skianis C. (2023). The impact of 6G-IoT technologies on the development of agriculture 5.0: a review. Electronics 12, 2651. 10.3390/electronics12122651

[B53] Reuveny R Thompson WR (2008). Uneven economic growth and the world economy's north–south stratification. Int. Stud. Quart. 52, 579–605. 10.1111/j.1468-2478.2008.00516.x

[B54] Senekane M. (2019). Differentially private image classification using support vector machine and differential privacy. Mach. Learn. Knowl. Extract. 1, 483–491. 10.3390/make1010029

[B55] Senekane M. Mafu M. Taele B. M. (2017). “Privacy-preserving quantum machine learning using differential privacy,” in 2017 IEEE AFRICON (Piscataway, NJ: IEEE), 1432–1435.

[B56] Singh S. K. Azzaoui A. E. Salim M. M. Park J. H. (2020). Quantum communication technology for future ICT-review. J. Inform. Proc. Syst. 16, 1459–1478.

[B57] Steane A. (1998). Quantum computing. Rep. Prog. Phy. 61, 117. 10.1088/0034-4885/61/2/002

[B58] Tarasov P. A. Isaev E. A. Grigoriev A. A. Morgunov A. F. (2020). The utilization of perspective quantum technologies in biomedicine. J. Phy. Conf. Ser. 1439, 012040. 10.1088/1742-6596/1439/1/012040

[B59] The Nobel Prize (1979). The Nobel Prize in Physics 1979. Available online at: https://www.nobelprize.org/prizes/physics/1979/summary/ (accessed June 20, 2023).

[B60] Thew R Jennewein T Sasaki M (2019). Focus on quantum science and technology initiatives around the world. Quantum Sci. Technol 5, 010201. 10.1088/2058-9565/ab5992

[B61] Torgler B. (2020). “Big data, artificial intelligence, and quantum computing in sports,” in 21st Century Sports: How Technologies Will Change Sports in the Digital Age, Cham: Springer Nature. 153–173.

[B62] UNGA (2019). Political Declaration of the High-Level Political Forum on Sustainable Development Convened Under the Auspices of the General Assembly. United Nations. Available online at: https://undocs.org/en/A/HLPF/2019/L.1 (accessed August 6, 2023).

[B63] Unwin PT. (2009). ICT4D: Information and Communication Technology for Development. Cambridge: Cambridge University Press.

[B64] Ur Rasool R. Ahmad H. F. Rafique W. Qayyum A. Qadir J. Anwar Z. (2023). Quantum computing for healthcare: a review. Future Internet 15, 94. 10.3390/fi15030094

[B65] Walsham G. (2017). ICT4D research: reflections on history and future agenda. Inform. Technol. Dev. 23, 18–41. 10.1080/02681102.2016.1246406

[B66] Wang R. Blagrave K. (2021). Using Quantum Computing Optimization to Maximize Agricultural Field Efficiency and Improve Crop Network Connectivity. Available online at: http://quantummindag.org/research.html

[B67] Xuereb A. (2022). Quantum Diplomacy: Rebalancing the Power Dynamic through Emerging Technologies. 10.1126/scidip.ade6826

[B68] Yin P. Takeuchi Y. Zhang W. H. Yin Z. Q. Matsuzaki Y. Peng X. X.. (2020). Experimental demonstration of secure quantum remote sensing. Phy. Rev. Appl. 14, 014065. 10.1103/PhysRevApplied.14.014065

